# Dysbiosis of gut microbiota and its correlation with dysregulation of cytokines in psoriasis patients

**DOI:** 10.1186/s12866-021-02125-1

**Published:** 2021-03-08

**Authors:** Xinyue Zhang, Linjing Shi, Ting Sun, Kun Guo, Songmei Geng

**Affiliations:** Department of Dermatology, Northwest Hospital, The Second Hospital Affiliated to Xi’an Jiaotong University, 157 Xiwu Road, Xi’an City, 710004 Shaanxi Province China

**Keywords:** Psoriasis, Gut microbiome, 16S, Microbiota, Cytokines

## Abstract

**Background:**

Psoriasis is an inflammatory skin disease associated with multiple comorbidities and substantially diminishes patients’ quality of life. The gut microbiome has become a hot topic in psoriasis as it has been shown to affect both allergy and autoimmunity diseases in recent studies. Our objective was to identify differences in the fecal microbial composition of patients with psoriasis compared with healthy individuals to unravel the microbiota profiling in this autoimmune disease.

**Results:**

We collected fecal samples from 30 psoriasis patients and 30 healthy controls, sequenced them by 16S rRNA high-throughput sequencing, and identified the gut microbial composition using bioinformatic analyses including Quantitative Insights into Microbial Ecology (QIIME) and Phylogenetic Investigation of Communities by Reconstruction of Unobserved States (PICRUSt). Our results showed that different relative abundance of certain bacterial taxa between psoriasis patients and healthy individuals, including Faecalibacterium and Megamonas, were increased in patients with psoriasis. It’s also implicated that many cytokines act as main effect molecules in the pathology of psoriasis. We selected the inflammation-related indicators that were abnormal in psoriasis patients and found the microbiome variations were associated with the level of them, especially interleukin-2 receptor showed a positive relationship with Phascolarctobacterium and a negative relationship with the Dialister. The relative abundance of Phascolarctobacterium and Dialister can be regard as predictors of psoriasis activity. The correlation analysis based on microbiota and Inflammation-related indicators showed that microbiota dysbiosis might induce an abnormal immune response in psoriasis.

**Conclusions:**

We concluded that the gut microbiome composition in psoriasis patients has been altered markedly and provides evidence to understand the relationship between gut microbiota and psoriasis. More mechanistic experiments are needed to determine whether the differences observed in gut microbiota are the cause or consequences of psoriasis and whether the relationship between gut microbiota and cytokines was involved.

**Supplementary Information:**

The online version contains supplementary material available at 10.1186/s12866-021-02125-1.

## Background

Psoriasis is an immune-mediated, genetic disease manifesting lesions with or without joint involvement. Due to the increasing prevalence and the complex pathogenesis, psoriasis has attracted worldwide attention in these years [1, 2]. Infection, genetic factors, and exceptional immunity may be involved in the pathogenesis of psoriasis. The viewpoint that the immune system is involved in the pathogenesis of psoriasis also has been widely accepted [3], especially Th17 cells play a crucial role in the pathogenesis and development of psoriasis [4]. Th17 cells, as well as Th1 cells and keratinocytes, secrete TNF-α, IFN-γ, IL-1β, IL-6, IL-12, IL-17A, IL-22, IL-23 participating in pathophysiologic processes of psoriasis [5] and current biological therapies as T cell-directed agents have demonstrated excellent efficacy in psoriasis. Among them, serum IL-6 and IL-22 levels were considered to be positively correlated with the severity of psoriasis inpatients [6, 7]. Furthermore, it was reported intestinal flora alteration could activate an abnormal immune response that ultimately leads to the development of psoriasis [8]. However, it has not been reported whether the gut mictobiota is correlated with the level of inflammatory factors, or whether the microecology dysbiosis correlated with the severity of psoriasis.

A large and complex community of beneficial microbes remain stable over a long period harbored in the human gut [9]. The stability of beneficial microbes is closely related to human health [10]. Studies have proven that the microbiome regulated the immune response in many physiological processes via interactions between innate immunity and acquired immunity [11, 12]. Recently, there has been growing interest in studies primarily focused on interactions between the gut microbiota and immune diseases, including psoriasis.

Several studies have identified gut microbiota dysbiosis as possible triggers or causes for recurrent episodes of psoriasis. Intestinal permeability was more frequently was reported in plaque psoriasis patients, and the bacterial DNA from the gut can be detected in patients’ blood [13]. This result explained the possible conditions that gut microbiota became one of the pathogenic factors of psoriasis. Genome sequencing showed that the presence of gut microbiota had potential effects on psoriasis [14–17]. They all showed that patients with psoriasis harbored the gut microbes were significantly different compared with healthy people. One of them pointed out that the microbiota dysbiosis in arthropathic psoriasis patients was similar to the patients with inflammatory bowel disease. Several organisms are virtually absent in these two diseases [14]. There was even evidence that manipulating the composition of the gut microbiota affected phenotypes of psoriasis [18]. However, current cross-secti studies could not determine whether the microbiome’s change is a cause or a result. Thus, further evidence, including consistency, specificity, timeliness, and biological plausibility, is needed to understand the relationship between gut microbes and the pathogenesis of psoriasis.

In the present study, we aimed to characterize the intestinal microbiota composition of patients with psoriasis and provide evidence for the hypothesis that the intestinal microbiota dysbiosis may play a vital role in the development of the psoriatic disease. Besides, we also collected laboratory indicators to analyze the correlation between clinical phenotypes and microbe species in the psoriasis group. An attempt was made to link the intestinal microbiota with the pathogenesis of psoriasis by immune-related inflammatory factors.

## Results

### The characteristics of participants

To explore the microbial composition of psoriasis patients in comparison with healthy controls, 60 fecal samples from 30 healthy controls and 30 psoriasis patients were collected. 20 males and 10 females were included in healthy controls and their mean age is (43.7 ± 13.21) years old. There were 20 males and 10 females in psoriasis group, with a mean age of (43.13 ± 13.79) years old. And their mean duration of the disease was (14.75 ± 11.32) years. According to the medical history and physical examinations, 18 of the 30 patients with psoriasis had the symptoms of pruritus. The skin lesions were found to be more than 50% in 18 of the 30 patients (Table 1).

**Table 1:** Characteristics of patients with psoriasis (*n* = 30) and healthy individuals (n = 30) included in this study

	Psoriasis	Healthy	P-value
Male/Female	20 / 10	20 / 10	1.000
Age (years)	43.13 ± 13.79	43.7 ± 13.21	0.871
Early-onset psoriasis (< 40 years)	25 / 30	–	–
Duration of the disease (years)	14.75 ± 11.32	–	–
Pustular psoriasis	6 / 30	–	–
Pruritus	18 / 30	–	–
Psoriasis area ≥ 50%	18 / 30	–	–

The biochemical examination results of psoriasis patients showed that the mean level of white blood cell (WBC) was 7.55 ± 0.52 × 10^9^/L, of alanine transaminase (ALT) was (21.95 ± 3.87) U/L, of interleukin-2R (IL-2R) was 678.61 ± 106.33 U/ml, of aspartate aminotransferase (AST) was (20.18 ± 1.90) U/L, of interleukin-8 (IL-8) was 45.07 ± 31.57 pg/ml, of Immunoglobulin A (Ig A) was 2.54 ± 0.26 g/L, of interleukin-6 (IL-6) was 10.91 ± 4.59 pg/ml, of total cholesterol (TC) was (3.99 ± 0.18) mmol/L, of complement 3 (C3) was 1.09 ± 0.03 g/L, of triglyceride (TG) was (1.62 ± 0.19) mmol/L, of high-density lipoprotein (HDL) was (1.02 ± 0.05) mmol/L, of low-density lipoprotein (LDL) was (2.68 ± 0.15) mmol/L, of fasting blood glucose (FBS) was (4.88 ± 0.16) mmol/L and of hypersensitive c-reactive protein (hsCRP) was (11.88 ± 4.72) mg/L, of Immunoglobulin E (Ig E) was 2.54 ± 0.26 IU/ml.

### Fecal microbiota analysis

**Fig. 1:**
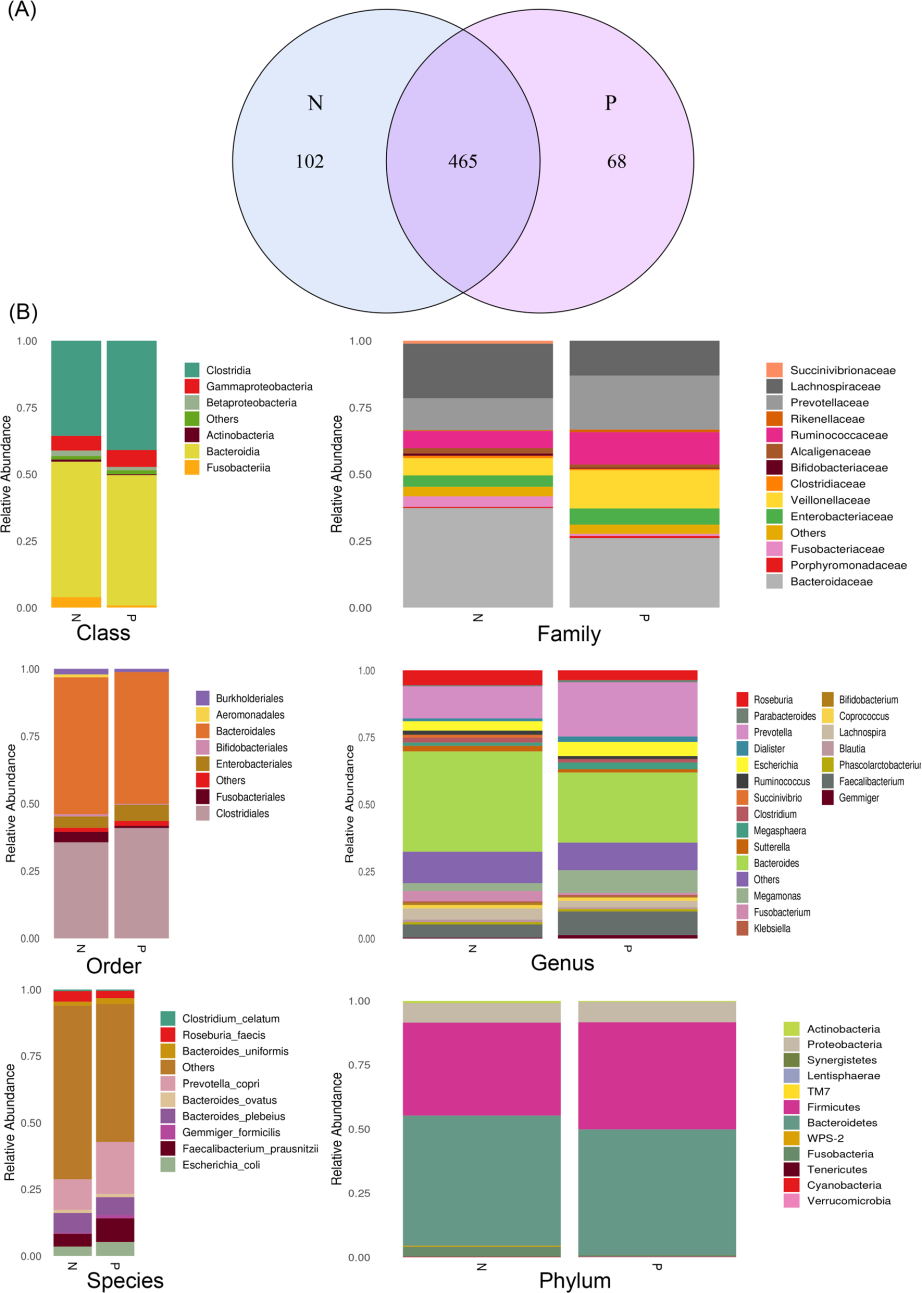
**a** Venn diagram showing the unique and shared operational taxonomic unit (OUT) in psoriasis (P) and healthy control group (N); **b** Comparison of fecal microbiota among psoriasis (P) and healthy control group (N). Relative abundance of the gut microbiota identified at class, family, order, genus, phylum and species level

We extracted Bacterial DNA from fecal samples and used 16S rRNA sequencing analyses. A total of 7656 518 single-end reads was generated from 60 samples clean reads and there was an average of 127,608 reads in one sample. A total of 635 OTUs were obtained, using Venn diagrams to visually show the number of shared and unique OTUs in different groups. The results showed that there were about 465 OTUs shared in the two groups, 102 and 68 OTUs in the healthy control group (N) and the psoriasis group (P), respectively (Fig. 1a). Then the OTUs sequence and Greengenes database were annotated, and the result showed that predominant microbes were included (Fig. 1b).

The OTU abundance curves of samples tend to plateau, showing that sample biodiversity was sufficiently covered with the applied sequencing depth (Supplement 1). The Alpha diversity box showed that there was no significant difference based on the Wilcox Test in the microbial diversity of psoriasis patients (P) and the healthy controls (N). The sobs index is 140 ± 50.824 and 150.1 ± 45.883, *P* = 0.34; Chao index is 162.91 ± 56.14 and 176.75 ± 45.51, *P* = 0.21; ace index is 162.58 ± 53.83 and 176.29 ± 45.81, *P* = 0.27; Shannon index is 2.55 ± 0.63 and 2.56 ± 0.65, *P* = 0.92; simpson index is 0.18 ± 0.11 and 0.20 ± 0.15, *P* = 0.81; coverage index is 1.00 ± 0.01 and 1.00 ± 0.01, *P* = 0.34 in healthy controls and psoriasis patients respectively (Supplement 2 and 3).

**Fig. 2:**
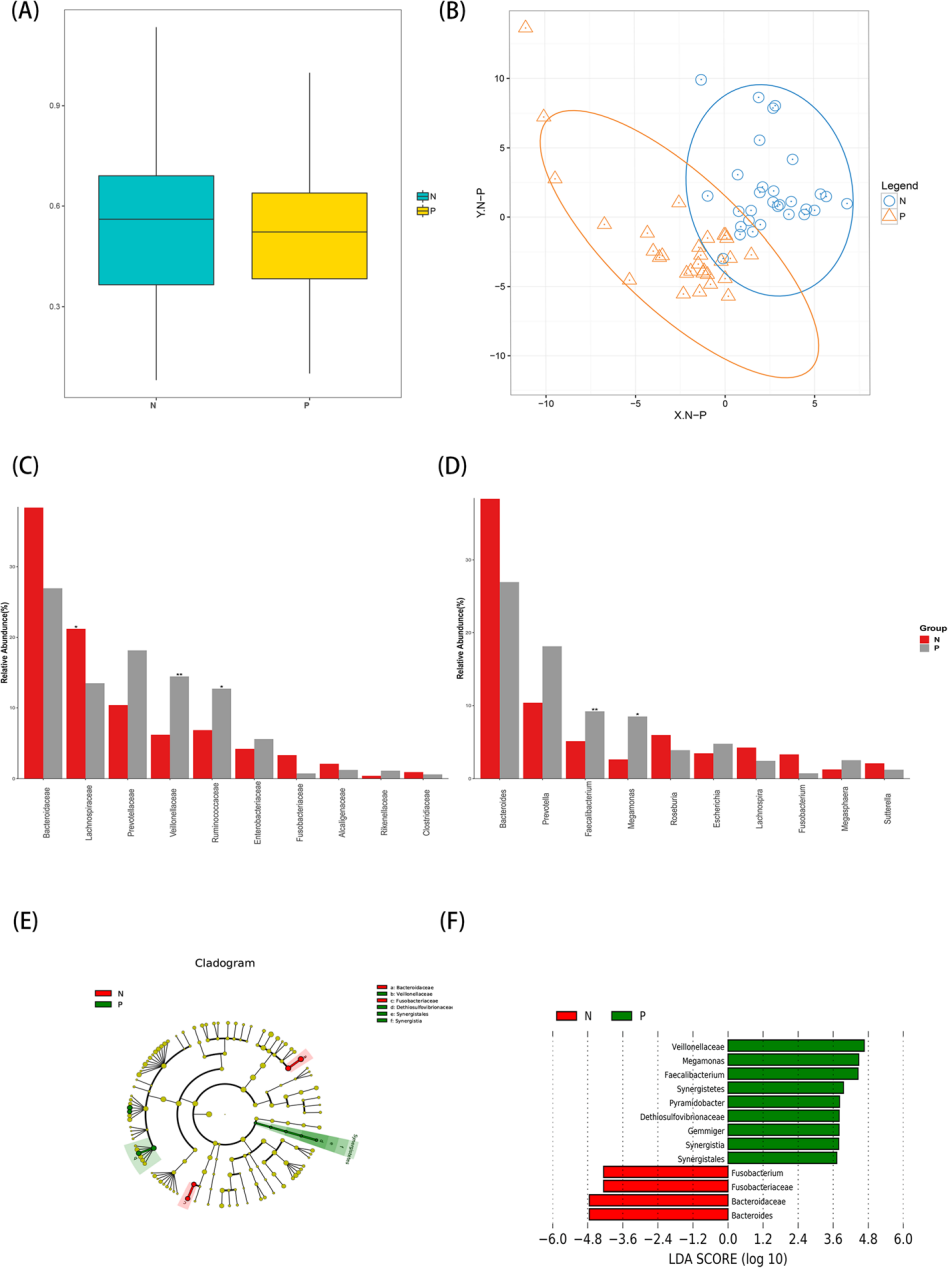
**a** Analysis of Beta diversity revealed significant differences between psoriasis group (P) and healthy control group (N) performed on the unweighted UniFrac (P<0.001). The results revealed a significant separation in the bacterial community composition between psoriasis patients and healthy individual; **b** PLS-DA plot of OTU. The horizontal axis and the vertical axis indicate the top 2 components. Each dot represents one sample and yellow dots stand for psoriasis group (P) and blue dots stand for healthy control group (N). It revealed significant clustering of psoriasis group (P) and healthy control group (N); **c** and **d** The dominant Family and Genus in the psoriasis group (P) and healthy control group (N). Histogram representation of the relative abundance (%) of the main taxa and the significant differences (*P*-values) observed between two groups. * 0.01 < P-value <= 0.05, ** 0.001 < = P-value <= 0.01; **e** and **f** The enriched taxa in psoriasis group (P) and control (N) fecal microbiota were represented in Cladogram. The central point represents the root of the tree (Bacteria), and each ring represents the next lower taxonomic level (phylum to genus: p, phylum; c, class; o, order; f, family; g, genus). The diameter of each circle represents the relative abundance of the taxon. The taxa with no significant differences were colored yellow, the red nodes represented the microbiota that played an important role in the healthy control group (N). The green node represents the microbiome that plays an important role in the psoriasis group (P) .And the most differentially abundant taxa between the psoriasis group (P) and healthy control group (N) which was generated from LEfSe analysis

Moreover, the β-diversity analyses showed distinct clustering (*P*-value < 0.001) between healthy controls and psoriasis patients, supporting the result that the gut microbiota composition was different in these two groups (Fig. 2a and b).

Next, we analyzed the gut bacterial communities of healthy controls and psoriasis patients. At the family and genus level, the microbial composition of psoriasis patients was significantly different from that of healthy controls. We showed the top 10 taxa in relative abundance at the family level, and the Wilcoxon test showed that Veillonellaceae and Ruminococcaceae were more abundant in psoriasis patients (*p* < 0.05). In contrast, Lachnospiraceae were less abundant in psoriasis patients (p < 0.05). Moreover, at the genus level, Faecalibacterium and Megamonas were more abundant in psoriasis patients compared with health controls (p<0.05) (Fig. 2c and d). We then conducted a LEfSe comparison of the gut microbiota between the control and psoriasis groups to explore the specific bacterial taxa associated with psoriasis development. The structure and predominant bacteria of the microbiota in the control and psoriasis groups were represented in a cladogram (Fig. 2e). The greatest difference in taxa from the phylum to the genus level was identified by an LDA score (Fig. 2f). The two taxa with the highest score in the psoriasis group were Veillonellaceae and Megamonas, consistent with the previous one.

Correlation between gut microbiota and clinical indices in psoriasis patients.

**Fig. 3:**
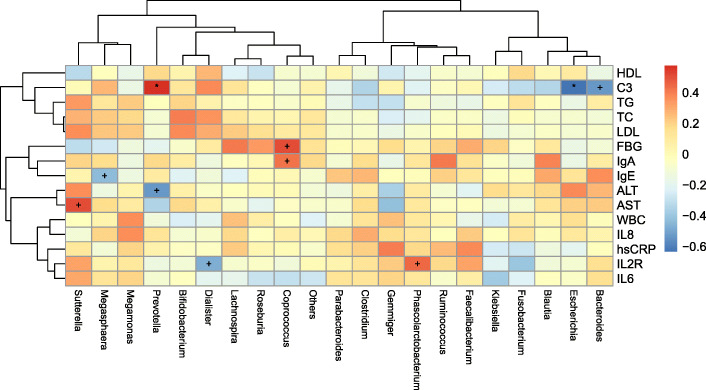
Spearman correlation analysis was performed to assess the correlation of clinical phenotypes with microbes at Genus level in the psoriasis group.* *P*-value<0.01, + P-value<0.001

Correlation analyses were used to investigate associations of key microbiota with clinical indices above (Fig. 3). Exact figures of these clinical indices were showed in Supplement 4. Complement 3 (C3) showed a negative relationship with Bacteroides (p<0.001) and Escherichia (p<0.01), but it showed a positive relationship with the Prevotella group (p<0.01). Coprococcus had a strong relationship with various clinical indices, and it showed a positive relationship with FBS (p<0.001) and IgA (p<0.001). Additionally, IL2R, which is abnormal in psoriasis patients, showed a positive relationship with Phascolarctobacterium (p<0.001) and a negative relationship with the Dialister group (p<0.001).

Different microbiota analysis between psoriasis vulgaris (PV) and pustular psoriasis (PP).

**Fig. 4:**
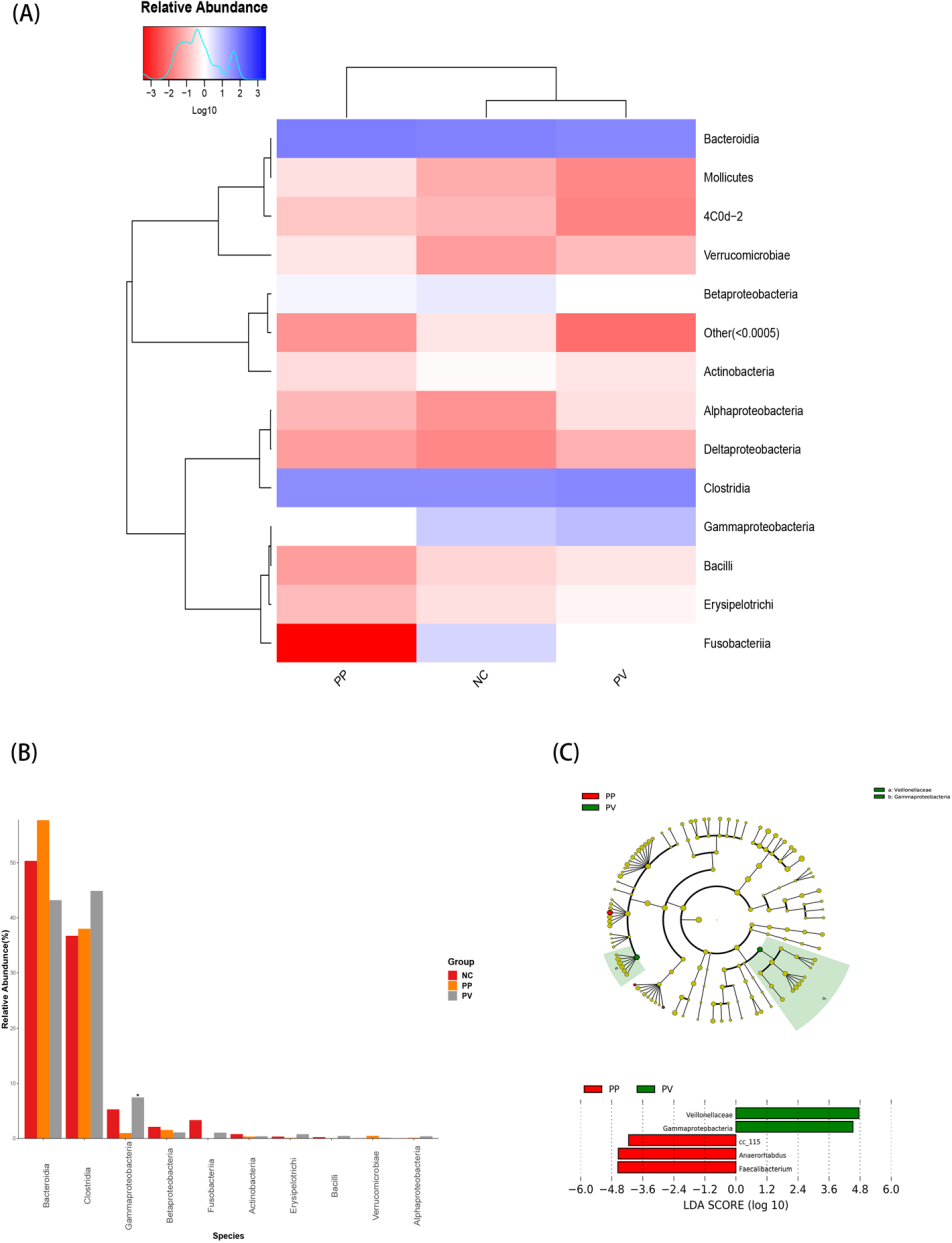
**a** Heat map based on the abundance ranks of the class level. Red and blue indicate high and low abundance, respectively. Hierarchical clustering (Euclidean distance, complete linkage) shows that psoriasis pustulosa group (PP) tends to be singled out against psoriasis vulgaris group (PV) and healthy control group (NC); **b** The dominant Class in the psoriasis pustulosa group (PP), psoriasis vulgaris group (PV) and healthy control group (NC). Histogram representation of the relative abundance (%) of the main taxa and the significant differences (*P*-values) observed between two groups. * 0.01 < P-value <= 0.05; **c** LefSe analysis was performed to identify differentially abundant taxa, which are highlighted on the phylogenetic tree in cladogram format and for which the LDA scores are shown. Red and green colors indicate an increase or decrease in taxa, respectively, in the psoriasis pustulosa group (PP) and psoriasis vulgaris group (PV)

To compare the microbiota communities of PV (24 cases) and PP (6 cases). Based on the abundance ranks of the class level, the heat map was demonstrated through R (v3.1.1) gplots. Hierarchical clustering (Euclidean distance, complete linkage) showed that the PV group and healthy control group (N) tended to cluster together, and the PP group is being singled out (Fig. 4a). Then we identified the presence of the top 10 taxa in relative abundance at the class level, and the Kruskal test showed that Gammaproteobacteria was relatively less abundant in the PP group than PV group and N group (all *P*-value<0.05) (Fig. 4b). LefSe analysis was performed to identify differentially abundant taxa in the PP group compared with the PV group (Fig. 4c). Consistent with our previous analysis, Gammaproteobacteria and Veillonellaceae had the largest LDA score indicating that Gammaproteobacteria and Veillonellaceae were consistently different between the PP group and PV group. In addition, the genus level Faecallibacterium and Anaerorhabdus were the most significant taxa in the PP group, which could help us distinguish PP from PV.

## Discussion

We used 16S rRNA gene profiling to elucidate the composition of the gut microbiota of psoriasis patients and evaluated the bacterial dysbiosis compared with healthy controls from the same geographic location.

We found no significant difference between the psoriasis patients and normal healthy people in alpha diversity, demonstrating that the levels of community species diversity and richness were similar in these two groups, consisting of Tan’s research in 2018 [15]. In other previous study, the lower microbial diversity was detected in patients with psoriasis [14, 17]. Also, differences in sequencing methodologies and the racial gap could lead to some of the differences observed between studies. In contrast to alpha diversity used in diversity within a sample, beta diversity is used to describe diversity between different samples and indicate the similarity of two samples. We found that the beta diversity revealed significant differences between the psoriasis patients (P) and the healthy controls (N), and two groups were clustered independently, which is consistent with the next analysis of gut microbiota composition. More specifically, the psoriasis patients collected in this study showed a microbiota profile characterized by a reduction in Lachnospiraceae but increased proportions of Veillonellaceae and Ruminococcaceae at the family level. At the genus level, Faecalibacterium and Megamonas showed a relative increase in microbial diversity of psoriasis patients. The increased proportion of Ruminococcaceae and Faecalibacterium was also detected in other researches [17, 18].

The depletion of Lachnospiraceae was also found in patients with primary Sjögren’s syndrome and primary Sjögren’s syndrome is a systemic inflammatory autoimmune disease that has similarities to psoriasis [19]. It was suggested a protective effect of Lachnospiraceae in the inflammatory process as its decreased abundance in these two Inflammatory disorders. Another study on amnestic mild cognitive impairment (aMCI) found that Lachnospiraceae was increased in aMCI subjests and their results represented Lachnospiraceae had beneficial effects on hosts [20]. It’s proved the relative abundance of Veillonellaceae is increased in Crohn’s disease patients in an early study [21]. Crohn’s disease is related to host pathways that indicated an underlying role for aberrant immune responses to the intestinal microbiota. The research on psoriatic arthritis pointed gut microbiota profile detected in psoriatic arthritis was similar to inflammatory bowel disease [22]. So we speculated that Veillonellaceae played a role in abnormal immunity related to psoriasis arthritis and even other psoriasis subgroups. We also found Megamonas was significantly higher in the psoriasis group. Megamonas was significantly correlated with systemic inflammatory cytokines and detected increased significantly in many other pathological processes, including HIV-1 Infection and obesity [23–25].

Previous studies showed the relative abundance of Faecalibacterium in gut microbiota was closely related to immune-regulation [26]. It’s also found that an increase in species belonging to this genus was associated with inflammatory diseases such as Crohn’s disease [27]. As for skin Diseases, Faecalibacterium subspecies have been shown to have a high relative abundance in infant eczema and atopic dermatitis, and these results indicated that Faecalibacterium might play a role in the disruption of barrier integrity and affection on the pro-inflammatory state in the gut [28, 29]. Bacteroides, Proteobacteria, Actinobacteria, and Akkermansia did not show otherness between groups detected abnormal in psoriasis patients in other studies [17, 18, 30]. As well, sample selection and sequencing analysis could lead to some differences observed between studies. Indeed, more research is needed to identify the differences and their significances.

One of the unique aspects of this study is correlation analysis between gut microbiota and inflammation-related indicators. The remarkable abnormality in relative abundance inclunding Lachnospiraceae, Veillonellaceae, and Ruminococcaceae from our results. These families are involved in butanoate metabolism and butyrate production [31, 32]. Butyrate, as a bacterial product, have been implicated in regulation of various inflammatory factors including IL-10, IL-6 and IL-18 [33]. Including butyrate-induced process, the mechanism of interaction between gut microbiota and inflammatory response still needs further study. It’s feasible to analyze the correlation between the serum level of inflammatory factors and intestinal microbial composition in psoriasis as abnormal cytokine production and enteric dysbacteriosis both involved in it [34].

We collected common biochemical indicators in patients with psoriasis, including cytokine test results representing the level of inflammation. IL-2R, IL-6, and IL-8 serum concentration in patients with psoriasis could be the predictor of disease severity and the marker of response to therapy [35–37]. Through the correlation analysis of clinical phenotypes with microbes, we found a strong positive correlation between Phascolarctobacterium and IL-2R. It’s indicated the increased abundance of Phascolarctobacerium could be regard as a pathogenic factor involved in the inflammatory response and a predictor of disease activity. Psoriasis is a chronic inflammatory skin disease in which effector T cells are increased in peripheral blood, and IL-2R as an activation marker of T cells is significantly elevated in psoriasis patients [35–37]. Microbiome dysbiosis has been proven to trigger several immune disorders through the activity of T cells and lead to an inflammatory process [38, 39]. The study showed that that treatment with broad-spectrum antibiotics in an imiquimod-induced inflammation psoriatic model in mice reduced the percentage of phenotypic skin thickness and active T cells [40]. Their results supported the relationship between microbiome dysbiosis and T cell-mediated inflammatory immune response. It’s indicated that Phascolarctobacterium was correlated significantly with the systemic inflammatory cytokines and psoriasis patients showed an increased presence of Faecalibacterium [24, 30]. Our results supported that cytokines and gut microbes were related in the occurrence of psoriasis and immunoreaction might be the bridge that connected them. We have found another interesting aspect that the level of C3 was in a negative correlation with Bacteroides in our research. It’s reported C3, and C4 levels were significantly higher in patients with psoriasis than in healthy controls [41], and the reduction of Bacteroides in psoriasis patients was also found by others [17]. The results suggest that an immune response associated with gut microbes may also activate complement involvement. The correlation between gut microbes and Inflammation-related indicators confirmed the involvement of gut microbes in the pathogenesis of psoriasis from one aspect and suggested the possible mechanism of the involvement of gut microbes in the pathogenesis of psoriasis.

To determine whether the gut microbiota in pustular psoriasis patients and psoriasis vulgaris patients is different, we compared the bacterial communities of patients with two types. The microbial compositions at the species level show that Faecalibacterium and Anaerorhabdus were significantly high in patients with pustular psoriasis. Pustular psoriasis is a heterogeneous group of inflammatory skin diseases genetically distinct from psoriasis vulgaris [42]. Our results indicated that pustular psoriasis was distinct from psoriasis vulgaris in gut bacterial communities and provided evidence for the differences in pathogenesis between pustular psoriasis and psoriasis vulgaris. However, other studies have found no significant differences between the four psoriasis subgroups [18]. Further studies are needed to determine the differences in the gut microbiota among the different psoriasis severity levels and subgroups.

In summary, we indicated different relative abundance for gut microbiota and suggested the microbial dysbiosis that existed in psoriasis patients. The correlation analysis with inflammatory cytokines and microbiota was further evidence that gut microbiota induced immunologic pathogenesis of psoriasis. Our results were consistent with previous research focused on psoriasis, even more autoimmune diseases. In a follow-up experiment, the pathogenic mechanism and effect of gut microbes on psoriasis still needed to be done. This entire endeavor is ultimately to achieve a microbiota-based therapeutic approach.

## Conclusion

Psoriasis is a chronic, immune-mediated skin disease that there is an increased incidence rate in recent years. Most advancements in psoriasis have been in its pathogenesis. It’s well accepted that abnormal immune responses mediated by cytokines are involved in the development of psoriasis, including TNF-α, IL-17, IL-6, etc. As a hotspot of research in recent years, the gut microbiota is related to both psoriasis and cytokines. However, whether the gut microbiota can be involved in the development of psoriasis, accompanied by the overproduction of inflammatory cytokines, still needs to be confirmed by many studies. Our manuscript shows that the gut microbiota profile of patients is associated with abnormal cytokine levels, based on the result that patients displayed a dysbiosis compared with normal controls. We also find that gut microbiota composition in patients with pustular psoriasis and psoriasis vulgaris has its characteristics. We predict that gut microbiota will play an essential role in the clinical diagnosis and treatment of psoriasis as therapeutic targets or biomarkers of psoriasis.

## Methods

### Study population

A total of 30 patients with a clinical diagnosis of psoriasis who were attending a dermatology outpatient clinic in the Second Affiliated Hospital of Xi ‘an Jiaotong University during one year from July 2018 to July 2019, among them 24 were diagnosed with psoriasis vulgaris (PV), 6 with pustular psoriasis (PP), and 30 age and sex-matched healthy individuals were included in the present study. Exclusion criteria included antibiotic usage during one month before the study, infectious disease, other allergic and autoimmune diseases, and cancer in both groups. This study’s protocol was approved by the local Ethical Committee of the Second Affiliated Hospital of Xi ‘an Jiaotong University. We informed all the volunteers about their participation in the study and received their signed informed consent.

### Fecal sample collection and DNA extraction

Fecal samples were collected and immediately stored at − 80 °C. Bacterial DNA was extracted from fecal samples with the QIAamp DNA Stool Mini Kit following product specification. Isolated DNA was stored at − 20 °C until sequencing.

### Sequencing

The PCR reaction system was composed of 30 ng genomic DNA samples and corresponding fusion primers, and PCR reaction parameters were set for PCR amplification. PCR amplification products were purified by Agencourt AMPure XP magnetic beads and dissolved in Elution Buffer. We used the Agilent 2100 Bioanalyzer to detect the fragment range and concentration of the library. The HiSeq platform was best suited to the size of the inserted fragment, so it was used for sequencing. Hyper-variable region V3 to V4 was selected using forward primer F515 (GTGCCAGCMGCCGCGG) and reverse primer: “E.coli 907–924” (CCGTCAATTCMTTTRAGT) to examine the bacterial composition. The QIIME [43], v1.7.0, the software was used to process the raw data files from the sequencer.

### Microbial profiling analysis and statistical analysis

The operational taxonomic unit (OTU) formation was performed using the QIIME reference optimal picking, and UPARSE [44], version 1.2.22q was used to perform the clustering. We selected a primer-specific version of the full GreenGenes 13.5 [45] as the reference database. The Mothur (version: 1.31.2) software was used to calculate the alpha diversity indices. The corresponding rarefaction curve and box/bar plots were drawn by software R. We used QIIME pipeline to calculate the beta distance between samples. Since the number of sequences could influence the beta diversity, samples were downsampled to the same sequencing depth, the minimum of sample size. LDA Effective Size (LEfSe) is a biomarker discovery and explanation tool which could be used for high-dimensional data. The statistical significance with biological consistency and effect size estimation were coupled by LEfSe [46]. The software used the non-parametric factorial Kruskal-Wallis (KW) sum-rank test to detect features with significant differential abundance with respect to the class of interest, then used LDA to estimate the effect size of each differentially abundant feature.

## Supplementary Information

**Additional file 1: Supplement 1.** OTU rank cruve; **Supplement 2.** Boxplot of alpha diversity; **Supplement 3.** Alpha diversity test result; **Supplement 4.** Clinical indices of psoriasis patients.

## Data Availability

The datasets generated for this study can be found in the SRA accession database: ID PRJNA646468. https://www.ncbi.nlm.nih.gov/bioproject/PRJNA646468.

## Abbreviations

OTU: Operational taxonomic units
LEfSe: Linear discriminant analysis effect size
LDA: Linear discriminant analysis
FBS: Fasting blood sugar
PV: psoriasis vulgaris
PP: Pustular psoriasis

## References

[1] Boehncke WH, Schon MP (2015). Psoriasis. LANCET.

[2] Griffiths C, van der Walt JM, Ashcroft DM, Flohr C, Naldi L, Nijsten T (2017). The global state of psoriasis disease epidemiology: a workshop report. Br J Dermatol.

[3] Chiricozzi A, Romanelli P, Volpe E, Borsellino G, Romanelli M. Scanning the Immunopathogenesis of Psoriasis. INT J MOL SCI. 2018;19(1):179. 10.3390/ijms19010179 PMC5796128 29316717

[4] Lowes MA, Russell CB, Martin DA, Towne JE, Krueger JG (2013). The IL-23/T17 pathogenic axis in psoriasis is amplified by keratinocyte responses. TRENDS IMMUNOL.

[5] Armstrong AW, Read C (2020). Pathophysiology, Clinical Presentation, and Treatment of Psoriasis: A Review. JAMA.

[6] Balato A, Schiattarella M, Di Caprio R, Lembo S, Mattii M, Balato N (2014). Effects of adalimumab therapy in adult subjects with moderate-to-severe psoriasis on Th17 pathway. J Eur Acad Dermatol Venereol.

[7] Luan L, Ding Y, Han S, Zhang Z, Liu X (2014). An increased proportion of circulating Th22 and Tc22 cells in psoriasis. CELL IMMUNOL.

[8] Benhadou F, Mintoff D, Schnebert B, Thio H (2018). Psoriasis and Microbiota: A Systematic Review. Diseases.

[9] Turnbaugh PJ, Ley RE, Hamady M, Fraser-Liggett CM, Knight R, Gordon JI (2007). The human microbiome project. NATURE.

[10] Coyte KZ, Schluter J, Foster KR (2015). The ecology of the microbiome: Networks, competition, and stability. SCIENCE.

[11] Holmes E, Li JV, Athanasiou T, Ashrafian H, Nicholson JK (2011). Understanding the role of gut microbiome-host metabolic signal disruption in health and disease. TRENDS MICROBIOL.

[12] Holmes E, Li JV, Marchesi JR, Nicholson JK (2012). Gut microbiota composition and activity in relation to host metabolic phenotype and disease risk. CELL METAB.

[13] Ramirez-Bosca A, Navarro-Lopez V, Martinez-Andres A, Such J, Frances R, Horga DLPJ (2015). Identification of Bacterial DNA in the Peripheral Blood of Patients With Active Psoriasis. JAMA DERMATOL.

[14] Scher JU, Ubeda C, Artacho A, Attur M, Isaac S, Reddy SM (2015). Decreased bacterial diversity characterizes the altered gut microbiota in patients with psoriatic arthritis, resembling dysbiosis in inflammatory bowel disease. ARTHRITIS RHEUMATOL.

[15] Tan L, Zhao S, Zhu W, Wu L, Li J, Shen M (2018). TheAkkermansia muciniphila is a gut microbiota signature in psoriasis. Exp Dermatol.

[16] Drago F, Ciccarese G, Indemini E, Savarino V, Parodi A (2018). Psoriasis and small intestine bacterial overgrowth. INT J DERMATOL.

[17] Hidalgo-Cantabrana C, Gomez J, Delgado S, Requena-Lopez S, Queiro-Silva R, Margolles A (2019). Gut microbiota dysbiosis in a cohort of patients with psoriasis. Br J Dermatol.

[18] Huang L, Gao R, Yu N, Zhu Y, Ding Y, Qin H (2019). Dysbiosis of gut microbiota was closely associated with psoriasis. SCI CHINA LIFE SCI.

[19] Cano-Ortiz A, Laborda-Illanes A, Plaza-Andrades I, Membrillo DPA, Villarrubia CA, Rodriguez CDMM, et al. Connection between the Gut Microbiome, Systemic Inflammation, Gut Permeability and FOXP3 Expression in Patients with Primary Sjogren's Syndrome. INT J MOL SCI. 2020;21(22):8733. 10.3390/ijms21228733 PMC7699261 33228011

[20] Liu P, Jia XZ, Chen Y, Yu Y, Zhang K, Lin YJ, et al. Gut microbiota interacts with intrinsic brain activity of patients with amnestic mild cognitive impairment. CNS Neurosci Ther. 2021;27(2):163–73. 10.1111/cns.13451 PMC7816203 32929861

[21] Gevers D, Kugathasan S, Denson LA, Vazquez-Baeza Y, Van Treuren W, Ren B (2014). The treatment-naive microbiome in new-onset Crohn's disease. CELL HOST MICROBE.

[22] Scher JU, Ubeda C, Artacho A, Attur M, Isaac S, Reddy SM (2015). Decreased bacterial diversity characterizes the altered gut microbiota in patients with psoriatic arthritis, resembling dysbiosis in inflammatory bowel disease. ARTHRITIS RHEUMATOL.

[23] Chiu C, Huang W, Weng S, Tseng H, Liang C, Wang W (2014). Systematic analysis of the association between gut Flora and Obesity through high-throughput sequencing and bioinformatics approaches. Biomed Res Int.

[24] Ling Z, Jin C, Xie T, Cheng Y, Li L, Wu N. Alterations in the Fecal Microbiota of Patients with HIV-1 Infection: An Observational Study in A Chinese Population. SCI REP-UK. 2016;6(1):30673. 10.1038/srep30673 PMC4967929 27477587

[25] Maya-Lucas O, Murugesan S, Nirmalkar K, Alcaraz LD, Hoyo-Vadillo C, Pizano-Zarate ML (2019). The gut microbiome of Mexican children affected by obesity. ANAEROBE.

[26] Miquel S, Martin R, Rossi O, Bermudez-Humaran LG, Chatel JM, Sokol H (2013). Faecalibacterium prausnitzii and human intestinal health. CURR OPIN MICROBIOL.

[27] Hansen R, Russell RK, Reiff C, Louis P, McIntosh F, Berry SH (2012). Microbiota of de-novo pediatric IBD: increased Faecalibacterium prausnitzii and reduced bacterial diversity in Crohn's but not in ulcerative colitis. AM J GASTROENTEROL.

[28] Abrahamsson TR, Jakobsson HE, Andersson AF, Bjorksten B, Engstrand L, Jenmalm MC (2012). Low diversity of the gut microbiota in infants with atopic eczema. J Allergy Clin Immunol.

[29] Zakostelska Z, Malkova J, Klimesova K, Rossmann P, Hornova M, Novosadova I (2016). Intestinal Microbiota Promotes Psoriasis-Like Skin Inflammation by Enhancing Th17 Response. PLOS ONE.

[30] Codoner FM, Ramirez-Bosca A, Climent E, Carrion-Gutierrez M, Guerrero M, Perez-Orquin JM, et al. Gut microbial composition in patients with psoriasis. Sci Rep. 2018;8(1):3812. 10.1038/s41598-018-22125-y PMC5830498 29491401

[31] Esquivel-Elizondo S, Ilhan ZE, Garcia-Pena EI, Krajmalnik-Brown R. Insights into Butyrate Production in a Controlled Fermentation System via Gene Predictions. MSYSTEMS. 2017;2(4):e00051–17. 10.1128/mSystems.00051-17 PMC5516221 28761933

[32] Louis P, Flint HJ (2017). Formation of propionate and butyrate by the human colonic microbiota. ENVIRON MICROBIOL.

[33] Singh N, Gurav A, Sivaprakasam S, Brady E, Padia R, Shi H (2014). Activation of Gpr109a, receptor for niacin and the commensal metabolite butyrate, suppresses colonic inflammation and carcinogenesis. IMMUNITY.

[34] Chiricozzi A, Guttman-Yassky E, Suarez-Farinas M, Nograles KE, Tian S, Cardinale I (2011). Integrative responses to IL-17 and TNF-alpha in human keratinocytes account for key inflammatory pathogenic circuits in psoriasis. J INVEST DERMATOL.

[35] Garshick MS, Barrett TJ, Wechter T, Azarchi S, Scher JU, Neimann A (2019). Inflammasome Signaling and Impaired Vascular Health in Psoriasis. Arteriosclerosisj Thromb Vasc Biol.

[36] Olejniczak-Staruch I, Narbutt J, Bednarski I, Wozniacka A, Sieniawska J, Kraska-Gacka M (2020). Interleukin 22 and 6 serum concentrations decrease under long-term biologic therapy in psoriasis. Postepy Dermatol Alergol.

[37] Owczarczyk-Saczonek A, Czerwinska J, Orylska M, Placek W (2019). Evaluation of selected mechanisms of immune tolerance in psoriasis. Postepy Dermatol Alergol.

[38] Littman DR, Pamer EG (2011). Role of the commensal microbiota in normal and pathogenic host immune responses. CELL HOST MICROBE.

[39] Honda K, Littman DR (2016). The microbiota in adaptive immune homeostasis and disease. NATURE.

[40] Zakostelska Z, Malkova J, Klimesova K, Rossmann P, Hornova M, Novosadova I (2016). Intestinal Microbiota Promotes Psoriasis-Like Skin Inflammation by Enhancing Th17 Response. PLOS ONE.

[41] Ozturk G, Erbas D, Gelir E, Gulekon A, Imir T (2001). Natural killer cell activity, serum immunoglobulins, complement proteins, and zinc levels in patients with psoriasis vulgaris. IMMUNOL INVEST.

[42] Bachelez H. Pustular Psoriasis: The Dawn of a New Era. Acta Derm Venereol. 2020. 10.2340/00015555-3388 PMC9128889 31971600

[43] Caporaso JG, Kuczynski J, Stombaugh J, Bittinger K, Bushman FD, Costello EK (2010). QIIME allows analysis of high-throughput community sequencing data. NAT METHODS.

[44] Edgar RC. UPARSE: highly accurate OTU sequences from microbial amplicon reads. Acta Derm Venereol. 2020;100(3):adv00034. 10.1038/nmeth.2604 23955772

[45] DeSantis TZ, Hugenholtz P, Larsen N, Rojas M, Brodie EL, Keller K (2006). Greengenes, a chimera-checked 16S rRNA gene database and workbench compatible with ARB. Appl Environ Microbiol.

[46] Segata N, Izard J, Waldron L, Gevers D, Miropolsky L, Garrett WS (2011). Metagenomic biomarker discovery and explanation. GENOME BIOL.

