# Supplementary material for: Dysbiosis of gut microbiota and its correlation with dysregulation of cytokines in psoriasis patients
Source: BMC Microbiol. 2021 Mar 8;21:78. doi: 10.1186/s12866-021-02125-1 (PMC7941898; doi:10.1186/s12866-021-02125-1)
Supplement: Supplementary file 1 — Additional file 1: Supplement 1. OTU rank cruve; Supplement 2. Boxplot of alpha diversity; Supplement 3. Alpha diversity test result; Supplement 4. Clinical indices of psoriasis patients. [file 12866_2021_2125_MOESM1_ESM.docx]

Supplementary material


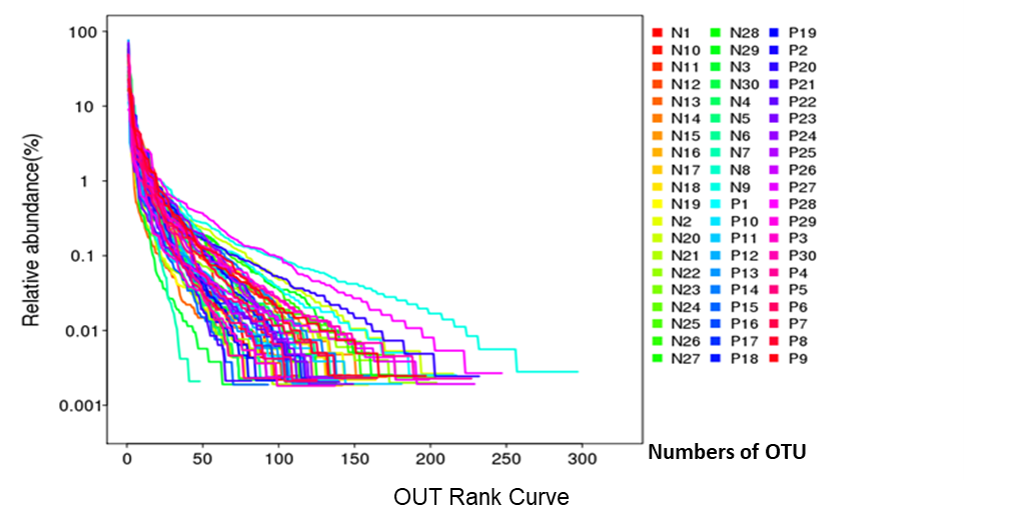


Supplement 1. The horizontal axis is the rank of OTU in decreasing order of abundance, while the vertical axis is the relative abundance of OTU. Each line represents one sample.


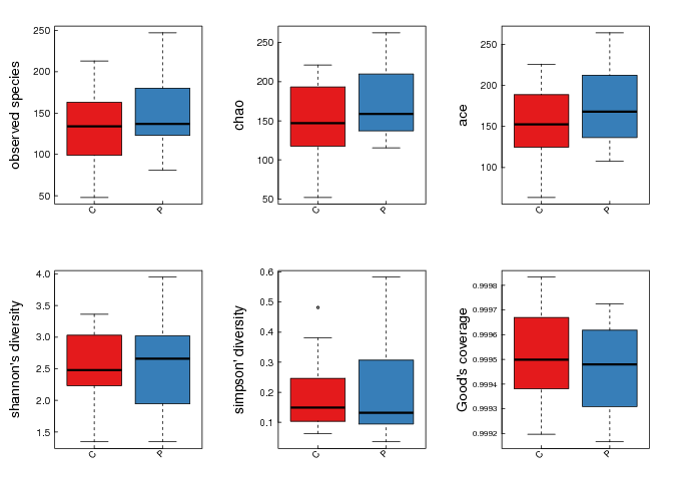


Supplement 2. Boxplot of alpha diversity. The figure shows the alpha diversity among groups. For all boxplots, black center lines represent the median and box edges are the first and third quartiles. The horizontal axis is group information and the vertical axis is the value of index. The red box represented healthy control group (C) and the blue box represented psoriasis patients group (P).

| **#Alpha** | **mean(C)** | **SD(C)** | **mean(P)** | **SD(P)** | **p-vaule** |
| --- | --- | --- | --- | --- | --- |
| **sobs** | **140** | **50.82356** | **150.1** | **45.88317** | **0.34398** |
| **chao** | **162.9168** | **56.13711** | **176.7536** | **45.51086** | **0.20784** |
| **ace** | **162.5852** | **53.8374** | **176.2936** | **45.81328** | **0.27289** |
| **shannon** | **2.54986** | **0.62968** | **2.56003** | **0.64969** | **0.92402** |
| **simpson** | **0.18491** | **0.11516** | **0.19554** | **0.1451** | **0.80868** |
| **coverage** | **0.99949** | **0.00021** | **0.99946** | **0.00017** | **0.33654** |

Supplement 3. Table 2.healthy control group (C) - psoriasis patients group (P) .Alpha diversity test result. The exact figures showed in boxplot of alpha diversity.

Supplement 4. Clinical indices of psoriasis patients

|  | WBC | ALT | AST | TC | TG | HDL | LDL | FBG | IgA | IgE | C3 | hsCRP | IL2R | IL6 | IL8 |
| --- | --- | --- | --- | --- | --- | --- | --- | --- | --- | --- | --- | --- | --- | --- | --- |
| P1 | 6.61 | 46 | 31 | 4.59 | 2.43 | 0.79 | 3.66 | 5.34 | 2.65 | 252 | 0.84 | 3.23 | 624 | 3.82 | 13.9 |
| P2 | 7.05 | 73 | 44 | 4.83 | 1.31 | 1.02 | 3.54 | 4.43 | 2.82 | 12.5 | 1.1 | 3.23 | 386 | 4.76 | 5 |
| P4 | 10.71 | 21 | 26 | 3.66 | 1.02 | 0.94 | 2.39 | 4.34 | 1.42 | 382 | 0.85 | 21.30 | 1971 | 11.8 | 23.6 |
| P5 | 5.62 | 17 | 14 | 4.19 | 2.55 | 1.19 | 2.74 | 3.87 | 1.46 | 188 | 1.07 | 3.23 | 246 | 3.81 | 7.27 |
| P7 | 10.96 | 16 | 15 | 3.99 | 1.26 | 0.85 | 2.75 | 4.68 | 1.57 | 8.61 | 0.95 | 3.23 | 501 | 9.77 | 11.3 |
| P9 | 10.36 | 6 | 11 | 2.45 | 0.65 | 0.65 | 1.54 | 4.54 | 1.92 | 181 | 1.15 | 105 | 1846 | 104 | 15.6 |
| P10 | 5.73 | 68 | 40 | 4.06 | 1.27 | 1.01 | 2.82 | 4.91 | 3.38 | 40.9 | 1.02 | 3.23 | 476 | 4.87 | 7.68 |
| P11 | 6.97 | 27 | 20 | 4.42 | 1.93 | 1.01 | 3.25 | 5.2 | 1.26 | 146 | 1.04 | 3.27 | 486 | 3.76 | 8.31 |
| P13 | 5.51 | 7 | 14 | 2.6 | 0.58 | 0.79 | 1.76 | 4.13 | 2.29 | 75.7 | 1.26 | 17.3 | 1399 | 8.18 | 22.6 |
| P14 | 8.69 | 15 | 15 | 4.1 | 1.99 | 0.77 | 3.08 | 5.27 | 3.55 | 205 | 1.18 | 21.6 | 526 | 4.81 | 8.08 |
| P15 | 11.46 | 26 | 20 | 4.29 | 1.23 | 1.16 | 3.02 | 7.36 | 2.53 | 19.2 | 1.07 | 7.91 | 390 | 5.45 | 9.37 |
| P17 | 13.5 | 22 | 25 | 3.76 | 1.12 | 0.74 | 2.68 | 4.71 | 5.31 | 968 | 0.98 | 27.3 | 1173 | 29.2 | 18.5 |
| P19 | 5.15 | 10 | 19 | 3.89 | 1.98 | 1.02 | 2.58 | 4.53 | 5.4 | 1310 | 1.07 | 3.23 | 1017 | 4.87 | 22 |
| P20 | 6.34 | 21 | 14 | 3.1 | 2.1 | 0.59 | 2.07 | 5.25 | 2.61 | 1230 | 1.01 | 3.23 | 789 | 2.48 | 5 |
| P21 | 6.05 | 28 | 29 | 4.67 | 2.76 | 1.18 | 3.03 | 4.62 | 1.49 | 49.9 | 1.09 | 3.23 | 375 | 4.66 | 6.07 |
| P22 | 7.69 | 11 | 12 | 3.47 | 1.1 | 1.55 | 1.72 | 4.61 | 4.58 | 908 | 1 | 3.23 | 353 | 3.76 | 8.25 |
| P23 | 7.22 | 8 | 13 | 3.72 | 0.81 | 1.34 | 2.25 | 4.99 | 2.31 | 567 | 1.28 | 3.23 | 351 | 8.06 | 5 |
| P25 | 6.36 | 12 | 14 | 2.87 | 0.58 | 1.05 | 1.61 | 4.69 | 1.21 | 34.7 | 0.91 | 3.23 | 258 | 4.31 | 707 |
| P27 | 8.37 | 13 | 20 | 3.69 | 3.02 | 0.77 | 2.27 | 3.92 | 1.94 | 12.2 | 1.21 | 3.23 | 481 | 6.71 | 39.4 |
| P28 | 7.13 | 10 | 15 | 5.54 | 1.02 | 1.42 | 3.73 | 4.8 | 1.68 | 18 | 1.26 | 7.9 | 241 | 2.5 | 23 |
| P29 | 4.29 | 15 | 15 | 4.11 | 1.11 | 1.31 | 2.69 | 5.14 | 1.67 | 8.14 | 1.14 | 3.23 | 370 | 4.73 | 7.51 |
| P30 | 4.4 | 11 | 18 | 5.87 | 3.9 | 1.21 | 3.9 | 6.06 | 2.8 | 13.9 | 1.44 | 7.88 | 670 | 3.77 | 17.2 |

Reference values and unit: White blood cell (WBC) 3.50-9.50 10^^9^/L; Alanine transaminase (ALT) 9-50 IU/L; Aspartate transaminase (AST) 15-40 IU/L; Total Cholesterol (TC) 3.3-5.8 mmol/L; Triglyceride (TG) 0.5-1.7 mmol/L; High-density lipoprotein (HDL) 0.8-1.8 mmol/L; Low-density lipoprotein (LDL) 2.3-3.3 mmol/L; Fasting blood-glucose (FBG) 3.3-6.0 mmol/L; IgA 0.70-4.00 g/L; IgE <100 IU/ml; C3 0.90-1.80 g/L; High sensitivity C reactive protein (hsCRP) 0-3.5 mg/L; Interleukin-2 Receptor (IL2R) 223-710 U/ml; Interleukin-6 (IL6) 0-3.4 pg/ml; Interleukin-8 (IL8) 0-14 pg/ml.
